# Graft-Derived Cell-Free DNA Quantification following Liver Transplantation Using Tissue-Specific DNA Methylation and Donor-Specific Genotyping Techniques: An Orthogonal Comparison Study

**DOI:** 10.3390/epigenomes7020011

**Published:** 2023-06-09

**Authors:** Daniel R. A. Cox, Tess McClure, Fan Zhang, Boris Ka Leong Wong, Adam Testro, Su Kah Goh, Vijayaragavan Muralidharan, Alexander Dobrovic

**Affiliations:** 1 Department of Surgery (Austin Precinct), University of Melbourne, Melbourne, VIC 3084, Australia; 2 HPB & Liver Transplant Surgery Unit, Department of Surgery, Austin Health, Melbourne, VIC 3084, Australia; 3 BEACON Biomarkers Laboratory, University of Melbourne, Melbourne, VIC 3084, Australia; 4 Liver Transplant Unit, Department of Gastroenterology & Hepatology, Austin Health, Melbourne, VIC 3084, Australia; 5 School of Cancer Medicine, La Trobe University, Melbourne, VIC 3084, Australia

**Keywords:** cell-free DNA, cfDNA, DNA methylation, liver Transplant*, donor-derived cfDNA, graft-derived cfDNA

## Abstract

*Background*: Graft-derived cell-free DNA (gdcfDNA) analysis has shown promise as a non-invasive tool for monitoring organ health following solid organ transplantation. A number of gdcfDNA analysis techniques have been described; however, the majority rely on sequencing or prior genotyping to detect donor-recipient mis-matched genetic polymorphisms. Differentially methylated regions of DNA can be used to identify the tissue-of-origin of cell-free DNA (cfDNA) fragments. In this study, we aimed to directly compare the performance of gdcfDNA monitoring using graft-specific DNA methylation analysis and donor-recipient genotyping techniques in a pilot cohort of clinical samples from patients post-liver transplantation. *Results*: 7 patients were recruited prior to LT, 3 developed early, biopsy-proven TCMR in the first 6 weeks post-LT. gdcfDNA was successfully quantified in all samples using both approaches. There was a high level of technical correlation between results using the two techniques (Spearman testing, r_s_ = 0.87, *p* < 0.0001). gdcfDNA levels quantified using the genotyping approach were significantly greater across all timepoints in comparison to the tissue-specific DNA methylation-based approach: e.g., day 1 post-LT median 31,350 copies/mL (IQR 6731–64,058) vs. 4133 copies/mL (IQR 1100–8422), respectively. Qualitative trends in gdcfDNA levels for each patient were concordant between the two assays. Acute TCMR was preceded by significant elevations in gdcfDNA as quantified by both techniques. Elevations in gdcfDNA, using both techniques, were suggestive of TCMR in this pilot study with a 6- and 3-day lead-time prior to histological diagnosis in patients 1 and 2. *Conclusions*: Both the graft-specific methylation and genotyping techniques successfully quantified gdcfDNA in patients post-LT with statistically significant concordance. A direct comparison of these two techniques is not only important from a technical perspective for orthogonal validation, but significantly adds weight to the evidence that gdcfDNA monitoring reflects the underlying biology. Both techniques identified LT recipients who developed acute TCMR, with several days lead-time in comparison to conventional diagnostic workflows. Whilst the two assays performed comparably, gdcfDNA monitoring based on graft-specific DNA methylation patterns in cfDNA offers major practical advantages over the donor-recipient genotyping, and hence enhances the potential to translate this emerging technology into clinical practice.

## 1. Background

T cell-mediated rejection (TCMR) can complicate patient recovery following liver transplantation (LT). Early TCMR typically presents in the first four to six weeks post-operatively, with reported incidence ranging from 20–60% of LT recipients [1,2]. Whilst many patients with TCMR respond well to pulsed steroid treatment, some develop steroid-resistant disease or chronic rejection which are associated with an increased risk of graft failure and mortality [3].

While conventional serum liver function tests (LFTs) are a highly *sensitive tool* for detecting graft injury; they lack *specificity* for its various causes and are poor predictors of TCMR or the degree its histological severity [4,5]. The gold-standard test for TCMR, therefore, remains percutaneous graft biopsy and histological tissue assessment [6]. Graft biopsy is unpleasant for patients and risks rare but serious peri-procedural complications including haemorrhage, bacteraemia, bile leak and death [7]. Clearly, accurate, non-invasive tests to diagnose or exclude TCMR would be highly desirable for patients and clinicians. 

Cell-free DNA (cfDNA) refers to nucleic acids in extracellular compartments including blood, urine and cerebrospinal fluid. Over the last decade, cfDNA analysis has demonstrated increasing utility across a range of medical applications, particularly in the oncology context [8,9,10]. Recently, graft-derived cell-free DNA (gdcfDNA) quantification has shown promise as a non-invasive tool for monitoring organ health, and in the diagnosis of TCMR following LT [11,12]. A number of molecular analysis techniques have been developed for gdcfDNA quantification following LT; however, the majority rely on sequencing or prior genotyping of the organ donor and recipient to identify mis-matched genetic polymorphisms to differentiate gdcfDNA from “background” cfDNA originating from other tissues [13]. 

A role for harnessing epigenetic signatures in cfDNA to improve molecular analysis has become increasingly apparent in recent years. Aberrant DNA methylation in cfDNA can be used to detect tumour-derived cfDNA [14,15]. Whilst the genomic sequence is generally preserved from cell-to-cell, DNA methylation patterns may be tissue-specific [16]. As such, appropriate differentially methylated regions (DMR) of DNA can be used to identify the tissue-of-origin of cfDNA fragments [17]. Leveraging tissue-specific methylation patterns in cfDNA offers an alternative method to quantify gdcfDNA following LT, that is yet to be thoroughly investigated in clinical studies [18,19].

In this prospective, observational cohort study we aimed to compare the efficacy of *longitudinal* gdcfDNA monitoring in LT recipients using *two orthogonal analytic methodologies* on the same set of samples, based on (a) tissue-specific methylation patterns in cfDNA and (b) donor-specific genetic polymorphisms. 

## 2. Materials and Methods 

This prospective, pilot, observational cohort study is reported in line with the “STROBE-ME” guidelines for reporting observational studies involving the use of molecular biomarkers (Supplementary Table S1) [20].

### 2.1. Patient Recruitment

Adult (>18 years) patients undergoing LT at a large Australian centre were invited to participate prior to their procedure. Exclusion criteria included: (i) multi-visceral transplantation, (ii) previous history of transplantation, and (iii) inability to provide written, informed consent.

### 2.2. Sample Collection, Processing and Storage

Blood samples (30 mL) were obtained from LT recipients pre-operatively and the organ donor at the time of graft procurement. Post-operatively, 30 mL blood samples were collected from the LT recipient on days 1, 3, 5, 7, 14, 28 and every 2 weeks following this, where possible. All samples were collected into EDTA-containing tubes and processed within 3 h of collection, as per recommended guidelines [21].

Samples underwent two-step centrifugation, initially at 800× *g* 10 min, ambient temperature (AT). Following this, the plasma fraction was isolated, with care to avoid disturbing the buffy coat layer. The plasma then underwent further centrifugation at 3000× *g* 10 min AT. The supernatant was collected without disturbing the cell pellet and was aliquoted into cryovials (Corning Inc, Corning, NY, USA) before storage at −80 °C. The buffy coat was also collected and stored in the same fashion. 

### 2.3. DNA Extraction and Bisulfite Modification

The QIAamp Circulating Nucleic Acid kit (Qiagen, Hilden, Germany) was used to extract cfDNA from 4 mL of plasma from each sample after thawing at AT (following the manufacturer’s instructions). Buffy coat DNA was extracted using the DNeasy Blood & Tissue Kit (Qiagen). DNA was eluted into an end volume of 50 μL of AVE buffer (Qiagen). 20 μL of the eluted DNA then underwent bisulfite modification using the EZ DNA Methylation-Lightning™ kit (Zymo Research, Irvine, CA, USA). The bisulfite-modified DNA was eluted into a final volume of 20 μL. Bisulfite-modified DNA was stored in DNA elution buffer at 4 °C before use.

### 2.4. gdcfDNA Quantification: Donor-Specific Genetic Polymorphism Technique

gdcfDNA quantification using the donor-specific genetic polymorphisms technique was performed in two steps:

#### 2.4.1. Genotyping for Identification of Informative Deletion/Insertion Genetic Polymorphisms

Buffy coat DNA obtained from the organ donors and recipients prior to LT, was genotyped using a panel designed to detect nine common deletion-insertion polymorphisms (DIPs) using high-resolution melting analysis (HRMA), as previously described, using the Mic qPCR Cycler (Bio Molecular Systems, Queensland, Australia) [22]. Results were analysed against control melt curves for three genotypes at each locus (insertion-insertion, deletion-deletion or deletion-insertion). A DIP allele that was present in the donor genome but absent in the organ recipient was selected for use for further gdcfDNA monitoring in post-operative samples. Supplementary Table S2 details the DIP targets employed and primer sets. The reaction mixture and PCR conditions are illustrated in Supplementary Table S3.

#### 2.4.2. Monitoring gdcfDNA Post-LT Using Insertion/Deletion Genetic Polymorphisms

Following identification of the donor-recipient DIP alleles, the Bio-Rad QX200 ddPCR™ system (Bio-Rad, Pleasanton, CA, USA) was used to quantify the presence of the donor-specific allele, as a marker of gdcfDNA, in post-operative plasma samples using a “probe-free” assay design. Supplementary Table S4 lists the primer panel sequences used in these experiments and Supplementary Table S5 details the reaction mixture and PCR cycling conditions used. 

### 2.5. gdcfDNA Quantification: Tissue-Specific DNA Methylation Technique

A set of methylation-independent primers, including seven *5′*—C—phosphate—G—*3′* (*CpG)* sites was designed (with an amplicon of 104 bp) based on a DMR identified by Gai et al. [23], that is highly methylated in liver in comparison to other tissues. Methylation-specific hydrolysis probes designed to hybridise to three methylated (HEX) or unmethylated (FAM) *CpG* sites were used to detect liver-specific cfDNA fragment methylation, as previously described [23]. 

A probe-based ddPCR assay was used to quantify liver-specific methylation in cfDNA fragments, as a marker of gdcfDNA, from post-operative plasma samples using the QX200 ddPCR™ system (Bio-Rad). Supplementary Table S6 presents the primer and probe sequences, reaction mixture and PCR conditions. 

### 2.6. Outcomes and Data Collection

All samples were analysed in duplicate for each assay and in batches (with care to avoid batch effects). Derived concentrations of gdcfDNA were normalised to copies/mL of plasma; the concentration of homozygote DIPs was halved to represent genomic content. A prospective database held by the Victorian Liver Transplant Unit was interrogated for clinical outcomes. The primary outcome of interest was the development of biopsy-proven early TCMR post-LT. gdcfDNA monitoring results did not influence clinical decision making. 

### 2.7. Statistical Analysis 

All data were complete and available for analysis. Median and interquartile range is presented for continuous variables, counts and percentages for categorical variables. The Mann-Whitney U test was used to compare continuous outcomes between the two groups. Spearman’s correlation coefficient (r_s_) was calculated to assess the rank correlation between values obtained using the two approaches. The analysis was performed using GraphPad Prism v9.3.1 for macOS (GraphPad Software, La Jolla, CA, USA).

## 3. Results 

### 3.1. Patient Outcomes

Seven patients undergoing LT were included, Table 1 summarises patients’ characteristics and clinical outcomes. Six patients were male, LT recipient age ranged from 21–60 years. Three patients developed early, biopsy-proven TCMR requiring treatment, all within 20 days of LT. Two of these patients (patients 2 and 7) had severe TCMR with histological rejection activity index (RAI) scores of 7–8/9 and 8–9/9 respectively [6]. Both patients were treated with pulsed IV methylprednisolone with an increase in tacrolimus dosage in patient 2. In patient 1, there was a gradual increment in serum gamma-glutamyl transferase (GGT) and alkaline phosphatase (ALP) levels post-operatively. Magnetic resonance cholangiopancreatography (MRCP) was performed at day 20 post-LT which ruled out biliary complications, and a graft biopsy on day 25 indicated mild TCMR (RAI = 3) which was treated with IV methylprednisolone. Patient 4 required a return to theatre on day 1 post-LT for concerns regarding hepatic artery thrombosis. The other patients had no major complications in the first 6 weeks post-transplantation. There were no instances of graft primary non-function or peri-operative mortality.

### 3.2. gdcfDNA Analysis

Sample timepoints ranged from day 0 (pre-operation) up to day 168 post-LT. Longitudinally collected samples for the first 6 weeks post-op were available for all of the patients except in patient 1 and 7, for whom available samples ranged up to day 28 and day 14 respectively. 

gdcfDNA was quantified in all samples using *both* molecular targets (tissue-specific DNA methylation and genetic polymorphisms). Table 2 illustrates the quantitative results. 

#### 3.2.1. gdcfDNA Results: Genetic Polymorphism Technique

**Figure 1:**
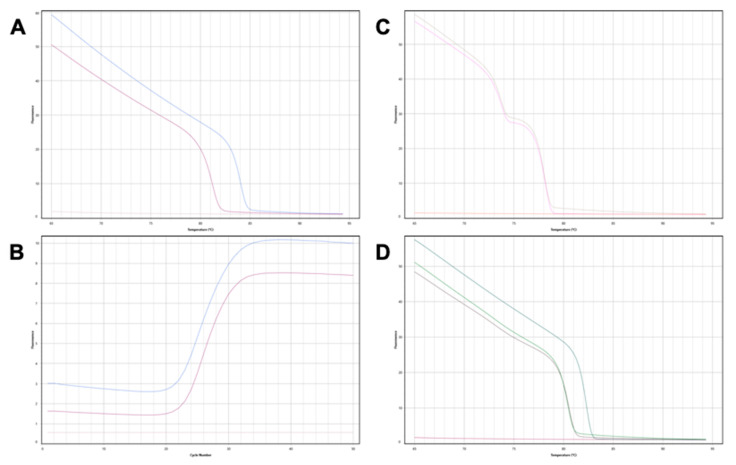
Genotyping donor and recipient DIPs for patient 7. (**A**) BTR 12 locus: the organ donor is homozygous ‘insertion-insertion’ (blue), the organ recipient is homozygous ‘deletion-deletion’ (purple), no template control (pink). The insertion allele forms a useful donor-recipient mismatch in this patient for gdcfDNA monitoring. (**B**) Corresponding PCR amplification curves to the melt curves seen in (**A**). (**C**) BTR 6 locus: the organ donor and organ recipient are both “heterozygotes” for the DIP at this locus. Genotyping this locus is therefore not useful for subsequent gdcfDNA quantification. (**D**) BTR 8 locus—both the organ donor and recipient are homozygotes for deletions at this DIP (green and purple). Homozygote insertion control (teal) curve included for comparison.

**Figure 2:**
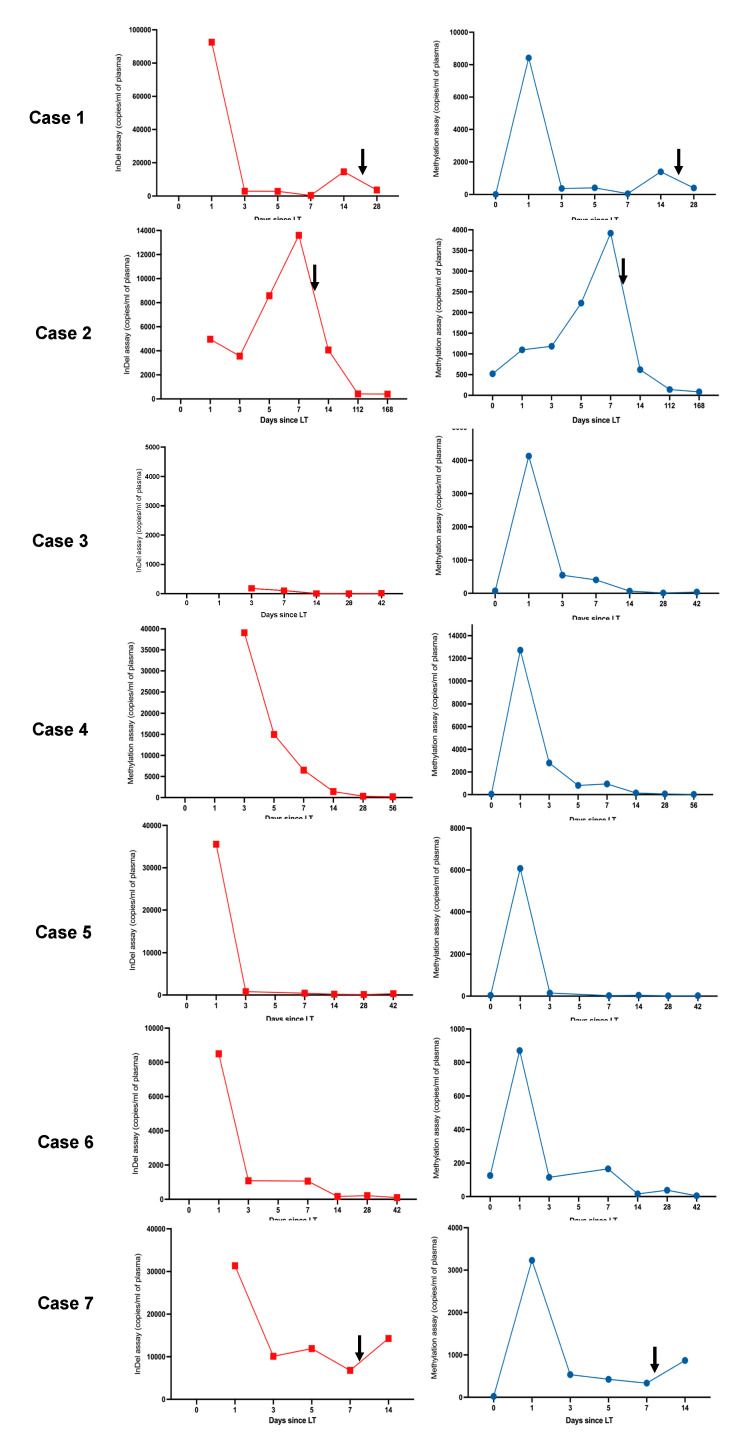
Parallel trends in gdcfDNA levels post-LT were seen when comparing both molecular diagnostic approaches across the cohort. gdcfDNA levels obtained using the DIPs genotyping technique are shown in red, results obtained using the tissue-specific DNA methylation approach are shown in blue. The black arrows indicate the timing of histological diagnosis of TCMR. ‘Day 0′ refers to pre-operative samples (analysis not possible for the DIPs approach as discussed in the text). The duration of monitoring (x-axis) differs between individuals. Note y-axis scales vary, peaks and nadirs illustrate changes in clinical status, not necessarily severity of disease. Trends in gdcfDNA between different individuals should not be compared using these graphs.

**Figure 3:**
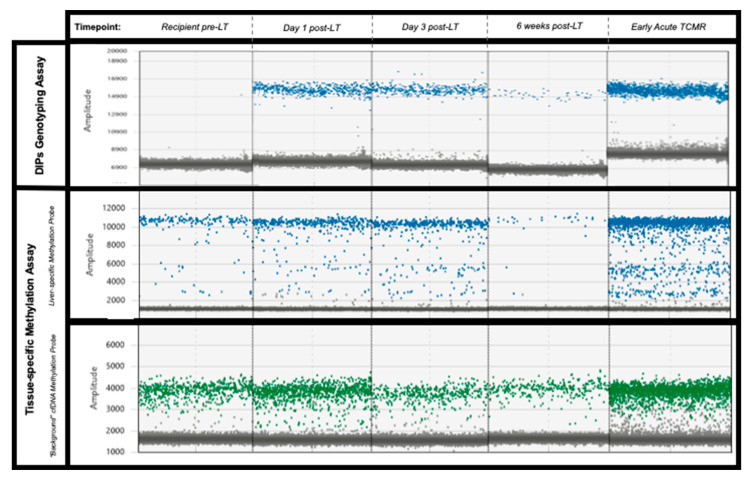
Illustrative examples of the digital PCR signal patterns seen in plasma samples from patient 2 using both approaches. No donor-specific DIPs are present in the organ recipient plasma sample pre-LT (top-left pane). Both assays demonstrate a similar decline in gdcfDNA levels post-LT. Levels of gdcfDNA increase significantly from baseline in cases of early TCMR (final column), using both of the assays. Key—Top row: genotyping technique, blue dots represent droplets positive for donor-specific DIP. Middle row: methylation-based technique, blue dots represent droplets with DNA containing liver-specific methylation signatures. Lowest row: methylation based-technique, “background” cfDNA with non-liver DNA methylation—green dots.

Informative, DIPs alleles were identified for each donor-recipient pair. In patients 1, 4 and 7 the grafts were homozygous for their respective donor-specific DIPs, the remainder were heterozygous. Figure 1 gives an illustrative example of the HRMA melt curves and amplification patterns seen in a donor-recipient pair. Digital PCR assays designed to amplify the donor-specific DIP loci successfully quantified gdcfDNA in the post-operative samples (Figure 2, in red). In each case, no amplification was demonstrated in DNA extracted from the recipient baseline sample, pre-LT, confirming the donor-specificity of the assays (Figure 3). gdcfDNA values ranged from 2 to 92,538 copies/mL of plasma. In patient 3 and 4, the day 1 sample was overly saturated with DNA, precluding meaningful analysis. 

gdcfDNA levels showed a stereotypic decline over time following LT in patients without early TCMR: median concentrations at day 1 = 22,038 copies/mL (IQR 8498–35,578) vs. day 7 = 752 copies/mL (IQR 189–5154). gdcfDNA levels, measured using the genetic polymorphism technique, became re-elevated in samples from all cases of early TCMR following the initial day 1 post-operative peaks, with a 6- and 3-day lead-time prior to histological diagnosis in patients 1 and 2 respectively (Figure 2). Using this technique, gdcfDNA levels increased by 98%, 37% and 53% from the sample leading up to the diagnosis of TCMR in patients 1, 2 and 7 respectively. gdcfDNA levels did not rise in relation to the suspected hepatic arterial thrombosis and return to theatre in patient 4.

#### 3.2.2. gdcfDNA Results: Tissue-Specific DNA Methylation Technique

gdcfDNA was quantified in all samples using the technique based on liver-specific DNA methylation patterns in cfDNA. No PCR amplification was seen in the no template control or in DNA extracted from patient buffy coat, indicating suitable clinical specificity of the assay. gdcfDNA levels ranged from 5 to 12,719 copies/mL of plasma across the entire cohort. Unlike in the case of the genetic polymorphism assay, liver-derived cfDNA was able to be quantified in recipient’s pre-operative samples, as the technique is not specific to donor-recipient mismatch for monitoring organ health.

Levels of methylation-detected gdcfDNA showed similar trends to those seen using the DIP approach (Figure 2), while the absolute concentration values were significantly lower. Liver-derived cfDNA levels ranged from 7 to 519 copies/mL in recipients at baseline prior to LT (median concentration 40 copies/mL [IQR 22–126]). The fraction of liver-derived cfDNA in organ recipients ranged from 0.2–31% preoperatively (measured as a proportion of background cfDNA unmethylated at the target locus, i.e., cfDNA derived from non-liver tissues). The median concentration of liver-derived cfDNA in the organ donor plasma prior to organ procurement was 388 copies/mL (IQR 160–610), with a median methylated epiallelic fraction of 2%. These values compare with an expected liver-derived cfDNA fraction ranging from 0.48–2.0% in healthy adults [24]. Interestingly a significant outlier in terms of donor and recipient baseline gdcfDNA levels, patient 2, (31% liver-derived cfDNA in recipient and 13% in donor), developed the most significant episode of early TCMR post-LT with the highest peak gdcfDNA levels, following day 1 post-operatively, across the cohort. 

Again, a general trend in declining gdcfDNA levels was observed in patients whose recovery was not complicated by early TCMR: median concentration at day 1 = 5103 copies/mL (IQR 1687–11,058) vs. day 7 = 285 copies/mL (IQR 60–810). Longitudinal gdcfDNA monitoring using the tissue-specific DNA methylation approach also showed significant elevation in levels associated with the occurrence of TCMR, but not in the development of hepatic arterial thrombosis seen in patient 4 (Figure 2 and Figure 3). gdcfDNA levels rose in the TCMR cases to a similar degree seen using the genetic polymorphism approach: 96%, 43% and 62% in patients 1, 2 and 7 respectively. 

### 3.3. Comparative Analysis

**Figure 4:**
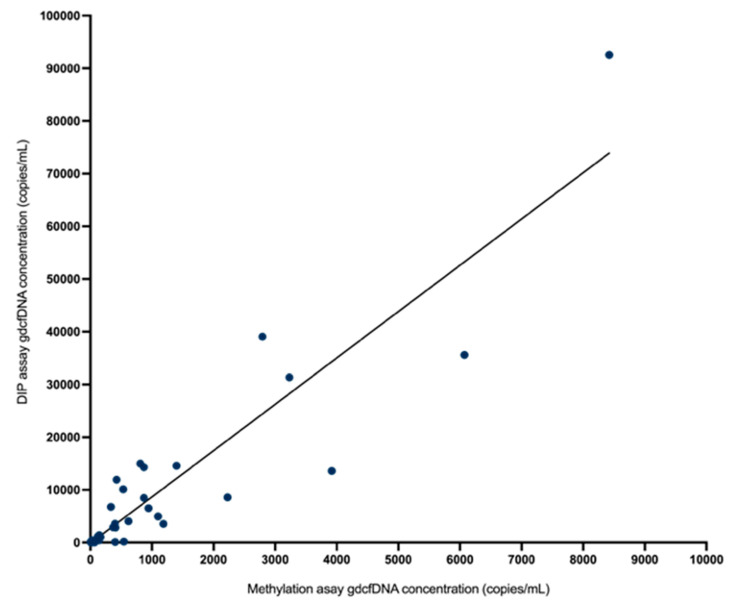
There was strong correlation in absolute quantification of gdcfDNA concentration by the two assays (r_s_ = 0.87, *p* < 0.0001) on Spearman correlation testing.

Monitoring of gdcfDNA levels using the two, orthogonal molecular analysis approaches showed qualitatively equivalent results in terms of the peaks and nadirs associated with initial graft recovery from operation and the occurrence of early TCMR (Figure 2). In terms of absolute quantification of gdcfDNA concentration, the two assays showed strong correlation (r_s_ = 0.87, p < 0.0001) on Spearman testing (Figure 4). The median fractional change in gdcfDNA from one timepoint to the next (excluding time-points prior to the onset of TCMR) for the DIPs assay was −185.38% and −246.18% for the methylation assay. Mean increase in gdcfDNA associated with early TCMR from sample time-points immediately prior to the TCMR event was +67% for the assay based on DIPs and +63% for the liver-specific methylation assay. 

## 4. Discussion

This study performed a head-to-head comparison of two parallel methodologies for gdcfDNA analysis, applied to the same sample set following transplantation. Both approaches performed well in demonstrating the kinetics of gdcfDNA release post-operatively. A peak in gdcfDNA levels was seen with both assays in the initial samples, followed by a gradual decline and equilibrium to baseline levels within the first two weeks of LT, in the cases of uncomplicated recovery. This initial dramatic release of gdcfDNA post-LT likely relates to substantial cellular death caused by the ischaemic-reperfusion injury relating to LT, and has been demonstrated in a number of other studies [25,26,27]. 

The methylation-based and genotyping-based assays both clearly identified patients who developed early TCMR, with spikes in gdcfDNA levels during the period of longitudinal monitoring (Figure 2). Whilst there was a general inverse trend in gdcfDNA levels over time post-LT, the incidence of TCMR was preceded by significant elevations in gdcfDNA concentrations in all cases. gdcfDNA monitoring anticipated biopsy-proven TCMR by 6- and 3-days in patients 1 and 2. Each assay showed specificity for the diagnosis of TCMR over other post-op complications, as in patient 4 who required a return to theatre for arterial thrombosis in the graft. Specificity for the diagnosis of TCMR over other post-LT complications, such as infection and cholestasis, has also been highlighted in other studies [28,29]. 

Both the genotyping assay and the assay based on liver-specific cfDNA methylation patterns performed comparably in monitoring graft health post-LT. Whilst a significant difference in absolute copy numbers of gdcfDNA was observed from analysis of the same samples using the two techniques, it is the relative changes in gdcfDNA release observed longitudinally—the “peaks and nadirs”—that are of translational interest clinically for monitoring organ heath. 

A direct comparison of these two techniques for monitoring gdcfDNA is not only important from an analytic perspective to support the technical performance of each approach by orthogonal validation (evidenced here by strong correlation in results across the two assays, r_s_ = 0.87) but is also significant in adding weight to evidence that gdcfDNA monitoring truly reflects the underlying biology. 

Whilst the two approaches for monitoring gdcfDNA performed technically well; the approach based on tissue-specific DNA methylation patterns has significant practical advantages enhancing its feasibility for translation into clinical care. Most importantly, the methylation-based assay circumvents the necessity for prior donor and recipient genotyping to enable monitoring. Genotyping is time consuming and significantly increases laboratory complexity; the technique based on donor-specific DIPs implemented here requires initial HRMA analysis using 18 different primers (Supplementary Table S2), other described techniques for genotyping prior to gdcfDNA monitoring have incorporated from 10 to 41 PCR assays [30,31]. Often an additional DNA extraction step to retrieve genomic DNA from the buffy-coat of the donor and recipient is also required, as described in this study.

Targeted next generation sequencing (NGS), is an alternative analysis approach that has been used to quantify gdcfDNA based on donor-recipient genotyping [28,32]. NGS circumvents the requirement for prior genotyping of donor and recipient as a large number of genetic polymorphisms can be measured in each sample coupled with bioinformatic pipelines capable of discerning donor-specific polymorphisms. However, sequencing-based approaches are time consuming and, given complexity, usually require samples to be shipped to a central laboratory—increasing turnaround times and decreasing geographic availability for testing. (e.g. [33]) gdcfDNA analysis using graft-specific DNA methylation patterns, as described here, avoids these issues through the use of a universal, single digital PCR assay that can be used in all cases. Digital PCR is a widely available technology meaning that sample analysis may be performed locally, enabling a clinically relevant same-day turnaround time, even for initial samples. 

Another advantage of the methylation-based gdcfDNA technique is that there is no requirement for donor blood or tissues for genotyping purposes. It is therefore also useful for monitoring graft health in cases of historic LT, where donor materials may not be available years later. gdcfDNA analysis using tissue-specific DNA methylation techniques is likely to be less susceptible to confounding related to peri-operative transfusion of blood products. In the case of gdcfDNA monitoring using a genotyping technique; gdcfDNA results may become unusable if an LT recipient is transfused with blood products from a blood donor with the same genotype as the organ donor [34]. As previously mentioned, in health, cfDNA in plasma has an approximate 2% contribution from the liver. Blood products derived from blood donors are likely to contain minimal amounts of liver-specific methylated sequences and are therefore less likely to confound gdcfDNA monitoring based on liver-specific cfDNA methylation analysis. 

In a previous cross-sectional study, we demonstrated that low levels of graft-specific methylation in cfDNA from a single plasma sample can be used stratify a low risk of TCMR in LT patients with abnormal liver enzymes [35]. Lehmann-Werman and colleagues (2018) reported gdcfDNA analysis in 6 patients sampled periodically post-LT using methylated ITIH4, IGF2R, and VTN cfDNA markers [19]. All of the patients developed biopsy-proven rejection, which was detectable in 5/6 patients using the cfDNA methylation panel. Gai et al. (2018) reported the quantification of gdcfDNA from 14 plasma samples derived from LT recipients post-transplantation with stable graft function, the mean epiallelic fraction of liver-derived cfDNA was 7.7% [23]. Whilst a wide number of different gdcfDNA quantification techniques based on mismatched donor/recipient genotyping have been described for monitoring graft health [12], clearly further investigation in larger study cohorts is required to investigate the translational utility of longitudinal gdcfDNA monitoring using cfDNA methylation markers in LT. 

Limitations of the methylation-based gdcfDNA quantification approach include that it requires an additional pre-analytical step prior to analysis; bisulfite modification of the extracted DNA. The bisulfite modification step takes approximately 2.5 h, and so requires much less time than genotyping and therefore does not preclude a same-day-turn around for analysis. However, bisulfite modification is associated with significant loss of DNA from the extracted analyte [36,37]. This is reflected in the decreased values in absolute copy number of gdcfDNA observed in this study using the methylation-based technique as compared with the genotyping assays. In other clinical settings, loss of DNA template prior to analysis may have significant effects on clinical utility, particularly when seeking to monitor rare epialleles. This effect is less important in the case of organ health monitoring post-LT, where clinical events (as shown in this study) as usually associated with a profound increase in gdcfDNA and thus reductions in sensitivity have less of an impact on interpretation of the underlying biology. The introduction of an additional pre-analytical variable (loss of DNA template) prior to analysis can be effectively monitored using a variety of internal control mechanisms, as has been shown in several studies [38,39].

Finally, the quantification of gdcfDNA using tissue-specific methylation patterns in cfDNA may be affected by the presence of heterogenous DNA methylation [40]. The “methylated” hydrolysis probe used in this study incorporates three CpG sites. Incorporation of the hydrolysis probe in the PCR reaction favours the situation where all 3 CpG epialleles are methylated. A DMR is a measure of specificity in DNA methylation. However, whilst DNA methylation in this region of the genome is highly specific to the liver, it may not be uniformly present in all liver cells. Therefore, heterogenous methylation in the 3 CpG sites within region of the target probe, might reduce the sensitivity of the biomarker. This sensitivity issue could be addressed through the development of a panel of tissue-specific DNA methylation markers for gdcfDNA to be quantified in each plasma sample. At this time, the addition of multiple targets would significantly increase laboratory complexity, turn-around time and expense. However, the recent introduction of multi-colour, multiplex digital PCR machines is likely to address this in the near future [41,42].

## 5. Conclusions

gdcfDNA levels were successfully monitored in this cohort of LT recipients using quantitative digital PCR approaches based on graft-specific DNA methylation and donor-recipient mismatched genotyping. Parallel and concordant trends in gdcfDNA were observed, in the same sample set, using the two techniques, with a high level of technical correlation between results. Both techniques identified LT recipients who developed acute TCMR, with a number of days lead-time in comparison to conventional diagnostic workflows. For successful translation into clinical practice, a gdcfDNA quantification technique must be accurate, economical, have a clinically-relevant turn-around time and, ideally, be simple to perform. Whilst the two assays performed comparably, gdcfDNA monitoring based on graft-specific DNA methylation patterns offers major practical advantages over techniques based on donor-recipient genotyping and hence enhances the potential to translate this emerging technology into clinical practice.

## Figures and Tables

**Table 1:** Patient characteristics and clinical outcomes.

Patient	Sex	Age (Years)	Indication for LT	Associated HCC?	TCMR (Day)	Details	Other Significant Post-Operative Complications
1	F	48	Alcohol and A1AT deficiency cirrhosis	No	Yes (20)	Mild rejection RAI 3.	Nil
2	M	30	PSC cirrhosis	No	Yes (10)	Moderate-Severe rejection RAI 7-8.	Nil
3	M	55	Acutely Decompensated HBV cirrhosis	No	No	N/A	Nil
4	M	54	HCV cirrhosis + HCC	Yes	No	N/A	Return to operating theatre day 1 for suspected HAT.
5	M	61	HCV cirrhosis + HCC	Yes	No	N/A	Nil
6	M	34	PSC cirrhosis	No	No	Nil	Nil
7	M	21	PSC cirrhosis	No	Yes (8)	Moderate-Severe RAI 8/9. No concerning changes in LFTs until Day 7. Serendipitous rapid access to ultrasound guided biopsy.	Nil

*Key*: A1AT—Alpha-1 antitrypsin, HAT—hepatic artery thrombosis, HCC—hepatocellular carcinoma, HBV—hepatitis B virus, HCV—hepatitis C virus, RAI—rejection activity index.

**Table 2:** Summary of gdcfDNA levels across the cohort as quantified using the two orthogonal techniques.

gdcfDNA Results		Patient 1	Patient 2	Patient 3	Patient 4	Patient 5	Patient 6	Patient 7
Pre-operative sample	DIPs	n/a	n/a	n/a	n/a	n/a	n/a	n/a
	Meth	7	519	75	40	37	126	22
	Meth%	0.2	31	1	1	4	12	3
Day 1 post-op	DIPs	92,538	4964	-	-	35,578	8498	31,350
	Meth	8422	1100	4133	12,719	6073	871	3231
	Meth%	80	41	296	508	300	50	111
Day 3 post-op	DIPs	2901	3561	180	39,050	815	1084	10,106
	Meth	371	1186	546	2795	146	115	536
	Meth%	24	114	38	51	21	21	44
Day 5 post-op	DIPs	2846	8580	-	14,988	-	-	11,921
	Meth	409	2228	-	811	-	-	426
	Meth%	17	28	-	8	-	-	19
Day 7 post-op	DIPs	349	13,599	105	6518	442	1062	6779
	Meth	52	3919	403	945	24	166	334
	Meth%	4	72	24	16	3	24	48
Day 14 post-op	DIPs	14,575	4070	6	1416	208	165	14,300
	Meth	1399	619	68	146	41	16	870
	Meth%	19	51	3	2	9	3	44
Day 28 post-op	DIPs	3589	-	2	330	132	214	-
	Meth	402	-	10	66	18	38	-
	Meth%	27	-	2	4	4	4	-
Day 42 post-op	DIPs	-	-	10	-	329	103	-
	Meth	-	-	37	-	23	5	-
	Meth%	-	-	6	-	7	1	-
Day 56 post-op	DIPs	-	-	-	188	-	-	-
	Meth	-	-	-	20	-	-	-
	Meth%	-	-	-	2	-	-	-
Day 112 post-op	DIPs	-	418	-	-	-	-	-
	Meth	-	141	-	-	-	-	-
	Meth%	-	12	-	-	-	-	-
Day 168 post-op	DIPs	-	404	-	-	-	-	-
	Meth	-	84	-	-	-	-	-
	Meth%	-	10	-	-	-	-	-

All values given in copies/mL of plasma. Key: - *result available. Meth%: the epiallelic fraction of liver-derived cfDNA,* i.e., *the proportion of liver-specific DNA methylation patterns in cfDNA against the background unmethylated target locus,* i.e., *cfDNA derived from all other tissues. %Meth = liver specific cfDNA methylation target concentration/ liver specific cfDNA methylation target + unmethylated ‘background’ cfDNA concentration at the target locus) × 100.*

## Data Availability

The data that support the findings of this study are available from the corresponding author upon reasonable request.

## Supplementary Materials

The following supporting information can be downloaded at: https://www.mdpi.com/article/10.3390/epigenomes7020011/s1, Table S1: STROBE-ME’ checklist for reporting observational studies involving molecular biomarkers.; Table S2: The targets and primer sets used to genotype donor and recipient buffy coat samples to identify donor-specific DIPs.; Table S3: The reaction mixture and PCR conditions used for HRMA to enable donor and recipient genotyping; Table S4: Primer panel used for quantitative digital PCR analysis following the DIPs genotyping technique.; Table S5:The reaction mixture and PCR cycling conditions used for quantitative digital PCR analysis in the donor and recipient genotyping technique; Table S6: Methylation-independent PCR primers, fluorescence probes, reaction mixture and PCR conditions used for tissue-specific DNA methylation-based gdcfDNA quantification.

## Abbreviations

A1AT—Alpha-1 antitrypsin deficiency, ALP—alkaline phosphatase, AT—ambient temperature, cfDNA—cell-free DNA, CpG—5’—C—phosphate—G—3’, DIPs—deletion-insertion polymorphisms, ddPCR—droplet-digital polymerase chain reaction, EDTA—ethylenediaminetetraacetic acid, GGT—gamma-glutamyl transferase, gdcfDNA—graft-derived cell-free DNA, HAT—hepatic artery thrombosis, HCC—hepatocellular carcinoma, HBV—hepatitis B virus, HCV—hepatitis C virus, HRMA—high-resolution melting analysis, IQR—interquartile range, IV—intravenous, LT—liver transplantation, MRCP—magnetic resonance cholangiopancreatography, PCR—polymerase chain reaction, PSC—primary sclerosing cholangitis, RAI—rejection activity index, TCMR—T-cell mediated rejection, TDS—three times daily, 95% CI—95% confidence interval. ITIH4—Inter-Alpha-Trypsin Inhibitor Heavy Chain 4. IGF2R—Insulin Like Growth Factor 2 Receptor. PTK2B—Protein tyrosine kinase 2 beta. VTN—Vitronectin.
