# Supplementary material for: Graft-Derived Cell-Free DNA Quantification following Liver Transplantation Using Tissue-Specific DNA Methylation and Donor-Specific Genotyping Techniques: An Orthogonal Comparison Study
Source: Epigenomes. 2023 Jun 9;7(2):11. doi: 10.3390/epigenomes7020011 (PMC10296814; doi:10.3390/epigenomes7020011)
Supplement: Supplementary file 1 [file epigenomes-07-00011-s001.zip › epigenomes-2336167-supplementary.pdf]

Graft-derived cell-free DNA quantification following liver transplantation using tissue-specific DNA methylation and donor-specific genotyping: an orthogonal comparison study

Cox DRA, McClure T, Zhang F, Wong BKL, Testro A, Goh SK, Muralidharan V, Dobrovic A.

Supplementary Data

Supplemental Table S1. ‘STROBE-ME’ checklist for reporting observational studies involving molecular biomarkers.

	Item No	STROBE Recommendation	STROBE-ME addition	Page
**Title and abstract**	1	(*a*) Indicate the study’s design with a commonly used term in the title or the abstract	ME-1 State the use of specific biomarker(s) in the title and/or in the abstract if they contribute substantially to the findings	1
		(*b*) Provide in the abstract an informative and balanced summary of what was done and what was found		3
**Introduction**				
Background/rationale	2	Explain the scientific background and rationale for the investigation being reported	ME-2 Explain in the scientific background of the paper how/why the specific biomarker(s) have been chosen, potentially among many others (e.g. others are studied but reported elsewhere or not studied at all)	5-6
Objectives	3	State specific objectives, including any prespecified hypotheses	ME-3 *A priori* hypothesis: if one or more biomarkers are used as proxy measures, state the *a priori* hypothesis on the expected values of the biomarker(s)	6
**Methods**				
Study design	4	Present key elements of study design early in the paper	ME-4 Describe the special study designs for molecular epidemiology (in particular, nested case/control and case/cohort) and how they were implemented	6
Biological sample collection		-	ME-4·1 Report on the setting of the biological sample collection; amount of sample; nature of collecting procedures; participant conditions; time between sample collection and relevant clinical or physiological endpoints	7,9
Biological sample processing		-	ME-4·2 Describe sample processing (centrifugation, timing, additives, etc.)	7
Biological sample storage		-	ME-4·3 Describe sample storage until biomarker analysis (storage, thawing, manipulation, etc.)	7
Biomarker biochemical characteristics		-	ME-4·4 Report the half-life of the biomarker and chemical and physical characteristics (e.g. solubility)	n/a
Setting	5	Describe the setting, locations, and relevant dates, including periods of recruitment, exposure, follow-up, and data collection		9
Participants	6	(*a*) *Cohort study*—Give the eligibility criteria, and the sources and methods of selection of participants. Describe methods of follow-up<br>*Case-control study*—Give the eligibility criteria, and the sources and methods of case ascertainment and control selection. Give the rationale for the choice of cases and controls<br>*Cross-sectional study*—Give the eligibility criteria, and the sources	ME-6 Report any habit, clinical condition, physiological factor or working or living condition that might affect the characteristics or concentrations of the biomarker	6-7

		and methods of selection of participants		
		(*b*) *Cohort study*—For matched studies, give matching criteria and number of exposed and unexposed<br>*Case-control study*—For matched studies, give matching criteria and the number of controls per case		
Variables	7	Clearly define all outcomes, exposures, predictors, potential confounders, and effect modifiers. Give diagnostic criteria, if applicable		10
Data sources/ measurement	8	For each variable of interest, give sources of data and details of methods of assessment (measurement). Describe comparability of assessment methods if there is more than one group	ME-8 Laboratory methods: report type of assay used, detection limit, quantity of biological sample used, outliers, timing in the assay procedures (when applicable) and calibration procedures or any standard used	8-9
Bias	9	Describe any efforts to address potential sources of bias		9
Study size	10	Explain how the study size was arrived at		Sample size determined by caseload and time
Quantitative variables	11	Explain how quantitative variables were handled in the analyses. If applicable, describe which groupings were chosen and why		10,11
Statistical methods	12	(*a*) Describe all statistical methods, including those used to control for confounding<br>(*b*) Describe any methods used to examine subgroups and interactions<br>(*c*) Explain how missing data were addressed<br>(*d*) *Cohort study*—If applicable, explain how loss to follow-up was addressed<br>*Case-control study*—If applicable, explain how matching of cases and controls was addressed<br>*Cross-sectional study*—If applicable, describe analytical methods taking account of sampling strategy<br>(*e*) Describe any sensitivity analyses	ME-12 Describe how biomarkers were introduced into statistical models	10
Validity/reliability of measurement and internal/external validation		-	ME-12·1 Report on the validity and reliability of measurement of the biomarker(s) coming from the literature and any internal or external validation used in the study	18
**Results**				
Participants	13	(a) Report the numbers of individuals at each stage of the study – e.g. numbers potentially eligible, examined for eligibility, confirmed eligible, included in the study, completing follow-up and analysed<br>(b) Give reasons for nonparticipation at each stage<br>(c) Consider use of a flow diagram	ME-13 Give reason for loss of biological samples at each stage	Table 1

Descriptive data	14	(a) Give characteristics of study participants (e.g. demographic, clinical and social) and information on exposures and potential confounders<br>(b) Indicate the number of participants with missing data for each variable of interest<br>(c) Cohort study – Summarize follow-up time (e.g. average and total amount)		Table 1
Distribution of biomarker measurement		-	ME-14·1 Give the distribution of the biomarker measurement (including mean, median, range and variance)	11-18
Outcome data	15	Cohort study – Report numbers of outcome events or summary measures over time<br>Case–control study – Report numbers in each exposure category or summary measures of exposure<br>Cross-sectional study – Report numbers of outcome events or summary measures		10
Main results	16	(a) Give unadjusted estimates and, if applicable, confounder-adjusted estimates and their precision (e.g. 95% confidence interval). Make clear which confounders were adjusted for and why they were included<br>(b) Report category boundaries when continuous variables were categorized<br>(c) If relevant, consider translating estimates of relative risk into absolute risk for a meaningful time period		11-18
Other analyses	17	Report other analyses done – e.g. analyses of subgroups and interactions and sensitivity analyses		18
**Discussion**				
Key results	18	Summarize key results with reference to study objectives		19-20
Limitations	19	Discuss limitations of the study, taking into account sources of potential bias or imprecision. Discuss both direction and magnitude of any potential bias	ME-19 Describe main limitations in laboratory procedures	19-24
Interpretation	20	Give a cautious overall interpretation of results considering objectives, limitations, multiplicity of analyses, results from similar studies and other relevant evidence	ME-20 Give an interpretation of results in terms of *a priori* biological plausibility	19-24
Generalizability	21	Discuss the generalizability (external validity) of the study results		19-24
**Other information**				
Funding	22	Give the source of funding and the role of the funders for the present study and, if applicable, for the original study on which the present article is based		25
Ethics		-	ME-22·1 Describe informed consent and approval from ethical committee(s). Specify whether samples were anonymous, anonymized or identifiable	24

Supplemental Table S2. The targets and primer sets used to genotype donor and recipient buffy coat samples to identify donor-specific DIPs.

Assay	Target DIP	Primers	Sequence (5'-3')
BTR02: Genetic polymorphism technique	UCSC hg38 co-ordinates - chr5: 65989041-65989066	Forward primer	GGTATGTGATAAGGAAGAGCCACT
		Reverse primer	TGCTCATTTTCTGGGTCGACACT
BTR03: Genetic polymorphism technique	UCSC hg38 co-ordinates - chr20: 34089222-34089247	Forward primer	CTCTTGAGATACATAGTTAAAGTAGACA
		Reverse primer	CCTGGACACTGAAAACAGGCAAT
BTR06: Genetic polymorphism technique	UCSC hg38 co-ordinates - chr5: 92978840-92978869	Forward primer	CTCTTGAGATACATAGTTAAAGTAGACA
		Reverse primer	CCTGGACACTGAAAACAGGCAAT
BTR08: Genetic polymorphism technique	UCSC hg38 co-ordinates - chr7: 30561807-30561825	Forward primer	CTCTTGAGATACATAGTTAAAGTAGACA
		Reverse primer	CCTGGACACTGAAAACAGGCAAT
BTR09: Genetic polymorphism technique	UCSC hg38 co-ordinates - chr10: 59679173-59679190	Forward primer	CCAAATTCAAGGAAACGGTGGTTCT
		Reverse primer	TCCGTGCTATTTCCTGCTGTTTCA
BTR12: Genetic polymorphism technique	UCSC hg38 co-ordinates - chr12: 119772141-119772155	Forward primer	AAGCAAAAGACCCAGCAATAGGAG
		Reverse primer	CCCTTGGTGTTTGTCCCACAGA
BTR16: Genetic polymorphism technique	UCSC hg38 co-ordinates - chr6: 48318749-48318835	Forward primer	AGCACGGATGTGAGGGCATCTT
		Reverse primer	GGCCAGGTCTATGAGGTCATCAA
BTR17: Genetic polymorphism technique	UCSC hg38 co-ordinates - chr5: 131390810-131390944	Forward primer	TGCTAGCCTCCCTGTTTGTCCT
		Reverse primer	GGTCCGGTGCAACTAAGTAATTCT
BTR18: Genetic polymorphism technique	UCSC hg38 co-ordinates - chr6: 6711451-6711525	Forward primer	GCTGCCATAACAAAGTACTGCAC
		Reverse primer	ACTTCCAGCCTCCAGCATGGT

Key - USCS hg38: University of California Santa Cruz human genome assembly 38.

Supplemental Table S3. The reaction mixture and PCR conditions used for HRMA to enable donor and recipient genotyping.

Reaction Mixture	PCR and Melting Conditions
Final Volume = 20 µL<br><br>1 PCR buffer, 2.5 mmol/L $MgCl^2$, 200 nmol/L forward primer, 200 nmol/L reverse primer, 20 ng of genomic DNA, 200 µmol/L of dNTPs (deoxynucleotide triphosphates), 5 µmol/L of SYTO 9 (In-vitrogen), 0.5 U of HotStarTaq (Qiagen) DNA polymerase, and PCR-grade water.	1 cycle of 95 °C for 15 min; 55 cycles of 95 °C for 10 s, 60 °C for 20 s, 72 °C for 30 s; 1 cycle of 97 °C for 1 min; and melt from 65 °C to 95 °C, rising 0.2 °C per second.

Supplemental Table S4. Primer panel used for quantitative digital PCR analysis following the DIPs genotyping technique.

Assay	Primers	Sequence (5'-3')
BTR02 Long:	Forward primer	GGTATGTGATAAGGAAGAGCCACT
	Reverse primer	GTCGACACTTTCTTCTTCGTTGTGA
BTR02 Short:	Forward primer	GGTATGTGATAAGGAAGAGCCACT
	Reverse primer	GTCGACACTTTCTTCTTCGTTGTGA
BTR03 Long:	Forward primer	GGTATGTGATAAGGAAGAGCCACT
	Reverse primer	TGCTCATTTTCTGGGTCGACACT
BTR03 Short:	Forward primer	TAGTTAAAGTAGACACGGCCAGAC
	Reverse primer	CCTGGACACTGAAAACAGGCAAT
BTR06 Long:	Forward primer	AAAGGGTTTCCTTGTTTTAGATAAGAGA
	Reverse primer	ATCCACACACAAAAGCAAAGCCAAAT
BTR06 Short:	Forward primer	AGGGTTTCCTTGTTACTTTCCATGTT
	Reverse primer	ATCCACACACAAAAGCAAAGCCAAAT
BTR08 Long:	Forward primer	AGTTCAGAGAAACATGCATTTCCAGA
	Reverse primer	AGACAGTGTTCCCATGCAAGAGT
BTR08 Short:	Forward primer	AGTTCAGAGAAACATGCATTTCCAGA
	Reverse primer	AGACAGTGTTCCCATGCAAGAGT
BTR09 Long:	Forward primer	CAAGGAAACGGTGGTTCTGTTTCT
	Reverse primer	TCCGTGCTATTTCCTGCTGTTTCA
BTR09 Short:	Forward primer	GGAAACGGTGGTTCTGTGCTGA
	Reverse primer	GACTAGATCTCCGTGCTATTTCCT
BTR12 Long:	Forward primer	AAGCAAAAGACCCAGCAATAGGAG
	Reverse primer	AAGGGGCGTGCCGTAGTTGATT
BTR12 Short:	Forward primer	AAGCAAAAGACCCAGCAATAGGAG
	Reverse primer	TGGTGTTTGTCCCACAGAAGGATT
BTR16 Long:	Forward primer	AGCACGGATGTGAGGGCATCTT

	Reverse primer	CGATGCATCCTATGTGCATGGC
BTR16 Short:	Forward primer	AGCACGGATGTGAGGGCATCTT
	Reverse primer	TCATCAAAAGACGATGCATGGCTG
BTR17 Long:	Forward primer	TGCTAGCCTCCCTGTTTGTCCT
	Reverse primer	GGTGCAACTAAGTAATTCTTTTCCCA
BTR17 Short:	Forward primer	TGCTAGCCTCCCTGTTTGTCCT
	Reverse primer	TCCGGTGCAACTAAGTAATTCTTTTATT
BTR18 Long:	Forward primer	GCTGCCATAACAAAGTACTGCAC
	Reverse primer	T GAGACGATGCATCTCTGGTTAAG
BTR18 Short:	Forward primer	GCTGCCATAACAAAGTACTGCAC
	Reverse primer	CTCCAGCATGGTGAGACTGGT

Supplemental Table S5. The reaction mixture and PCR cycling conditions used for quantitative digital PCR analysis in the donor and recipient genotyping technique.

Reaction Mixture	PCR and Melting Conditions
2 µL of eluted plasma cfDNA, 100 nmol/L of the forward and the reverse primer, 11 µL QX200 ddPCR EvaGreen Supermix (Bio-Rad), and PCR-grade water.	1 cycle of 95 °C for 5 min; 40 cycles of 95 °C for 30 s and 61 °C for 60 s; 1 cycle of 4 °C for 5 min; 1 cycle of 90 °C for 5 min and a brief hold at 4 °C

Supplemental Table S6. Methylation-independent PCR primers, fluorescence probes, reaction mixture and PCR conditions used for tissue-specific methylation-based gdcfDNA quantification.

Assay	Target	Primers	Sequence (5'-3')
Tissue-specific DNA methylation	*PTK2B*	Forward primer	TAGTTTTCGGAGTNGTTGTATATTTATTTG
	PTK2B	Reverse primer	CGAACTCCAACNACTACTCCTC
**Assay**	**Target**	**Probes**	**Sequence**
Tissue-specific DNA methylation	*Methylated PTK2B*	Methylated target probe	5'-FAM-TTGTCGTCGTTTCGGTT-MGB-3'
Tissue-specific DNA methylation	*Unmethylated PTK2B*	Unmethylated target probe	5'-HEX-TTTGTTGTTGTTTTGGTTTA-MGB-3'
**Reaction Mixture**		**ddPCR conditions**	
PCR-grade water, 11µL QX200 ddPCR Supermix for Probes (Bio-Rad), 450nM F primer, 450 nM R primer, 250nM Methylated Probe, 250nM Unmethylated Probe & 8 uL DNA template.		1 cycle of 95°C for 10 min; 45 cycles of 94°C for 15s then 56.5°C for 60 s; 98°C for 10 min; 4°C storage	
